# Three families of *q*-supercongruences modulo the square and cube of a cyclotomic polynomial

**DOI:** 10.1007/s13398-022-01338-x

**Published:** 2022-10-16

**Authors:** Victor J. W. Guo, Michael J. Schlosser

**Affiliations:** 1 School of Mathematics and Statistics, Huaiyin Normal University, Huai’an, 223300 Jiangsu People’s Republic of China; 2 Fakultät für Mathematik, Universität Wien, Oskar-Morgenstern-Platz 1, 1090 Vienna, Austria

**Keywords:** Basic hypergeometric series, Supercongruences, *q*-congruences, Cyclotomic polynomial, Gasper’s summation, Primary 33D15 Secondary 11A07

## Abstract

In this paper, three parametric *q*-supercongruences for truncated very-well-poised basic hypergeometric series are proved, one of them modulo the square, the other two modulo the cube of a cyclotomic polynomial. The main ingredients of proof include a basic hypergeometric summation by George Gasper, the method of creative microscoping (a method recently introduced by the first author in collaboration with Wadim Zudilin), and the Chinese remainder theorem for coprime polynomials.

## Introduction

More than one hundred years ago, in his second notebook, Ramanujan [19] mysteriously wrote 17 representations of $1/\pi$ (see [1, p. 352]), including for example,

1.1 $$\begin{aligned} \sum _{k=0}^{\infty }(6k+1)\frac{(\frac{1}{2})_k^3}{k!^3 4^k} =\frac{4}{\pi }, \end{aligned}$$

which he published in [19] later. Here, $(a)_n=a(a+1)\cdots (a+n-1)$ denotes the rising factorial. Van Hamme [21] numerically observed 13 amazing *p*-adic analogues of Ramanujan-type formulas, such as

1.2 $$\begin{aligned} \sum _{k=0}^{(p-1)/2}(6k+1)\frac{(\frac{1}{2})_k^3}{k!^3 4^k}&\equiv p(-1)^{(p-1)/2}\pmod {p^4}, \end{aligned}$$

where $p>3$ is a prime. But Van Hamme also said, “We have no real explanation for our observations." He himself proved three of these formulas. Supercongruences like (1.2) are now usually called Ramanujan-type supercongruences (see [24]). The proof of (1.2) was first given by Long [16] using hypergeometric evaluation identities. We refer the reader to [18] for historical remarks on Van Hamme’s supercongruences.

Recently, many classical supercongruences have been extended to supercongruences on rational functions in *q*, which we call *q-analogues of supercongruences*, or simply *q-supercongruences* (see [4, 5, 7–15, 17, 22, 23]). In particular, the first author [4] gave a *q*-analogue of (1.2) as follows: for odd *n*,

1.3 $$\begin{aligned}&\sum _{k=0}^{(n-1)/2}q^{k^2}[6k+1]\frac{(q;q^2)_k^2 (q^2;q^4)_k }{(q^4;q^4)_k^3} \nonumber \\&\qquad \quad \equiv (-q)^{(1-n)/2}[n] +\frac{(n^2-1)(1-q)^2}{24}(-q)^{(1-n)/2}[n]^3 \pmod {[n]\Phi _n(q)^3}. \end{aligned}$$

Here and throughout the paper, we adopt the standard *q*-notation: $[n]=1+q+\cdots +q^{n-1}$ is the *q*-*integer*; $(a;q)_n=(1-a)(1-aq)\cdots (1-aq^{n-1})$ is the *q-shifted factorial*, with the condensed notation $(a_1,a_2,\ldots ,a_m;q)_n=(a_1;q)_n (a_2;q)_n\cdots (a_m;q)_n$; and $\Phi _n(q)$ denotes the *n*-th *cyclotomic polynomial* in *q*, which may be given by

$$\begin{aligned} \Phi _n(q)=\prod _{\begin{array}{c} 1\leqslant k\leqslant n\\ \gcd (k,n)=1 \end{array}}(q-\zeta ^k), \end{aligned}$$

where $\zeta$ is an *n*-th primitive root of unity.

The cyclotomic polynomials $\Phi _n(q)$ are irreducible over the set of integers ${\mathbb {Z}}$, and, for any positive integer *n*, we have

1.4 $$\begin{aligned} \prod _{s>1,\;s\mid n}\Phi _s(q)=[n], \end{aligned}$$

which is a property we implicitly make use of in this paper.

Very recently, the present authors [9] proved the following result: Let *d* and *r* be odd integers satisfying $d\geqslant 3$, $r\leqslant d-4$ (in particular, *r* may be negative) and $\gcd (d,r)=1$. Let *n* be an integer such that $n\geqslant (d-r)/2$ and $n\equiv -r/2\pmod {d}$. Then

1.5 $$\begin{aligned} \sum _{k=0}^{n-1}[2dk+r]\frac{(q^r;q^d)_k^d}{(q^d;q^d)_k^d}q^{d(d-r-2)k/2} \equiv 0 \pmod {\Phi _{n}(q)^3}. \end{aligned}$$

In this paper, we consider the $r=d-2$ case of the left-hand side of (1.5) (in this case the condition $r\leqslant d-4$ for (1.5) is violated), and shall prove the following *q*-supercongruence.

### Theorem 1.1

Let $d\geqslant 3$ be an odd integer and let $n\equiv 1\pmod {d}$ be a positive integer. Then

1.6 $$\begin{aligned}&\sum _{k=0}^{n-1}[2dk+d-2]\frac{(q^{d-2};q^d)_k^d}{(q^d;q^d)_k^d} \nonumber \\&\qquad \quad \equiv \frac{[(d-2)n](q^{d-2},q^d;q^d)_{(d-2)(n-1)/d}}{(q,q^{d-1};q^d)_{(n-1)/d}(q^2,q^d;q^d)_{(2n-2)/d}^{(d-3)/2}} q^{-((d-1)^2+(d-3)^2n)(n-1)/(2d)} \pmod {\Phi _{n}(q)^3}. \end{aligned}$$

Further, the present authors [8] proved the following *q*-supercongruence: Let *d* and *r* be odd integers satisfying $d\geqslant 3$, $r\leqslant d-4$ (in particular, *r* may be negative) and $\gcd (d,r)=1$. Let *n* be an integer such that $n\geqslant d-r$ and $n\equiv -r\pmod {d}$. Then

1.7 $$\begin{aligned} \sum _{k=0}^{n-1}[2dk+r]\frac{(q^r;q^d)_k^d}{(q^d;q^d)_k^d}q^{d(d-r-2)k/2} \equiv 0 \pmod {\Phi _{n}(q)^3}. \end{aligned}$$

Note that in [8, Conjecture 3] it was even conjectured that the *q*-supercongruence (1.7) is true modulo $\Phi _n(q)^4$ for $d\geqslant 5$, which still remains open.

In this paper, we consider the $r=d-2$ case of the left-hand side of (1.7), and shall establish the following result.

### Theorem 1.2

Let $d\geqslant 3$ be an odd integer and let $n\equiv 2\pmod {d}$ be a positive integer. Then

1.8 $$\begin{aligned}&\sum _{k=0}^{n-1}[2dk+d-2]\frac{(q^{d-2};q^d)_k^d}{(q^d;q^d)_k^d} \nonumber \\&\quad \equiv -\frac{(q^d;q^d)_{(dn-n-d+2)/d}(1+q^{n^2(d-1)/2}) }{(1-q)(q^d;q^d)_{(n-2)/d}^{d-1}} q^{-d{(dn-n+2)/d\atopwithdelims ()2}} \pmod {\Phi _{n}(q)^2}. \end{aligned}$$

Further, the present authors (see [6, Theorem 1] and [7, Theorem 4.2]) proved the following result: Let *d*, *r*, *n* be integers satisfying $d\geqslant 3$, $r\leqslant d-2$ (in particular, *r* may be negative), and $n\geqslant d-r$, such that *d* and *r* are coprime, and $n\equiv -r\pmod {d}$. Then

1.9 $$\begin{aligned} \sum _{k=0}^{M}[2dk+r] \frac{(q^r;q^d)_k^{2d}}{(q^d;q^d)_k^{2d}}q^{d(d-1-r)k} \equiv 0 \pmod {[n]\Phi _{n}(q)^3}, \end{aligned}$$

where $$M=(dn-n-r)/d$$ or $$M=n-1$$.

In this paper, we consider the $r=d-1$ case of the left-hand side of (1.9), and shall build the following *q*-supercongruence.

### Theorem 1.3

Let $d\geqslant 3$ be an integer and let $n\equiv 1\pmod {d}$ be a positive integer. Then

1.10 $$\begin{aligned}&\sum _{k=0}^{M}[2dk+d-1]\frac{(q^{d-1};q^d)_k^{2d}}{(q^d;q^d)_k^{2d}} \nonumber \\&\quad \equiv [(d-1)n]\frac{(q^{d-1},q^{d};q^d)_{(d-1)(n-1)/d}}{(q,q^d;q^d)_{(n-1)/d}^{d-1}}q^{-((d-2)n+d)(d-1)(n-1)/(2d)}\pmod {[n]\Phi _n(q)^2}, \end{aligned}$$

where $$M=(d-1)(n-1)/d$$ or $$M=n-1$$.

Note that (1.10) is also true for $d=2$. See [4, 10] for a generalization of this case modulo $[n]\Phi _n(q)^3$.

In this paper we employ the method of creative microscoping, recently devised by the first author in collaboration with Zudilin [11], to prove Theorems 1.1–1.3. Here we appropriately introduce a parameter *a* (such that the series satisfies the symmetry $a\leftrightarrow a^{-1}$) into the terms of the series and prove that the congruences in Theorems 1.1 and 1.3 hold modulo $\Phi _n(q)$, modulo $1-aq^n$, and modulo $a-q^n$. Thus, by the Chinese remainder theorem for coprime polynomials, the congruences hold modulo the product $\Phi _n(q)(1-aq^n)(a-q^n)$. By letting $a= 1$ the congruences are established modulo $\Phi _n(q)^3$. By showing that the congruence in (1.9) also holds modulo $\Phi _s(q)$ for any divisor $s>1$ of *n*, we are able to lift the congruence modulo $\Phi _n(q)^3$ to the claimed congruence modulo $[n]\Phi _n(q)^2$. This again is justified by appealing to the Chinese remainder theorem for coprime polynomials, in combination with (1.4). For the congruence in Theorem 1.2, we similarly give a parametric generalization modulo $(1-aq^n)(a-q^n)$.

Our paper is arranged as follows: in Sect. 2, we list some tools we require in our proof of Theorems 1.1–1.3. These include a lemma on a simple *q*-congruence modulo a cyclotomic polynomial $\Phi _n(q)$, and a very-well-poised Karlsson–Minton type summation by Gasper of which we require some special cases. In Sect. 3, we first prove a parametric generalization of Theorem 1.1 involving the insertion of different powers of the parameter *a* in the respective *q*-shifted factorials. Afterwards we show how Theorem 1.1 can be deduced from this parametric generalization. We prove Theorems 1.2 and 1.3 similarly in Sects. 4 and 5, respectively.

## Preliminaries

We need the following result, which was first given by the present authors in [7, Lemma 2.1] (see also [9, Lemma 1]).

### Lemma 2.1

Let *d*, *m* and *n* be positive integers with $m\leqslant n-1$. Let *r* be an integer satisfying $dm\equiv -r\pmod {n}$. Then, for $0\leqslant k\leqslant m$ and any indeterminate *a*, we have

$$\begin{aligned} \frac{(aq^r;q^d)_{m-k}}{(q^d/a;q^d)_{m-k}} \equiv (-a)^{m-2k}\frac{(aq^r;q^d)_k}{(q^d/a;q^d)_k} q^{m(dm-d+2r)/2+(d-r)k} \pmod {\Phi _n(q)}. \end{aligned}$$

If $\gcd (d,n)=1$, then the above *q*-congruence also holds for $a=1$.

We will also use a very-well-poised Karlsson–Minton type summation of Gasper [2, Eq. (5.13)] (see also [3, Ex. 2.33 (i)]):

2.1 $$\begin{aligned}&\sum _{k=0}^\infty \frac{(a,q\sqrt{a},-q\sqrt{a},b,a/b,d,e_1,aq^{n_1+1}/e_1,\dots , e_m,aq^{n_m+1}/e_m;q)_k}{(q,\sqrt{a},-\sqrt{a},aq/b,bq,aq/d,aq/e_1,e_1q^{-n_1}, \dots ,aq/e_m,e_mq^{-n_m};q)_k}\left( \frac{q^{1-N}}{d}\right) ^k\nonumber \\&\qquad \quad =\frac{(q,aq,aq/bd,bq/d;q)_\infty }{(bq,aq/b,aq/d,q/d;q)_\infty } \prod _{j=1}^m\frac{(aq/be_j,bq/e_j;q)_{n_j}}{(aq/e_j,q/e_j;q)_{n_j}}, \end{aligned}$$

where $n_1,\dots ,n_m$ are non-negative integers, $N=n_1+\cdots +n_m$, and the convergence condition $|q^{1-N}/d|<1$ is necessary if the series does not terminate. The terminating $d=q^{-N}$ case of (2.1) reduces to

2.2 $$\begin{aligned}&\sum _{k=0}^N\frac{(a,q\sqrt{a},-q\sqrt{a},b,a/b,e_1,aq^{n_1+1}/e_1,\dots , e_m,aq^{n_m+1}/e_m,q^{-N};q)_k}{(q,\sqrt{a},-\sqrt{a},aq/b,bq,aq/e_1,e_1q^{-n_1}, \dots ,aq/e_m,e_mq^{-n_m},aq^{N+1};q)_k}q^{k} \nonumber \\&\qquad \quad =\frac{(q,aq;q)_N}{(bq,aq/b;q)_N}\prod _{j=1}^m \frac{(aq/be_j,bq/e_j;q)_{n_j}}{(aq/e_j,q/e_j;q)_{n_j}}. \end{aligned}$$

Note that an elliptic extension of (2.2) was provided by Rosengren and the second author [20, Eq. (1.7)].

In particular, taking $b\rightarrow \infty$ we get the following summation formula:

2.3 $$\begin{aligned}&\sum _{k=0}^N\frac{(a,q\sqrt{a},-q\sqrt{a},e_1,aq^{n_1+1}/e_1,\dots , e_m,aq^{n_m+1}/e_m,q^{-N};q)_k}{(q,\sqrt{a},-\sqrt{a},aq/e_1,e_1q^{-n_1}, \dots ,aq/e_m,e_mq^{-n_m},aq^{N+1};q)_k} \nonumber \\&\qquad \quad =\frac{(q,aq;q)_N q^{{n_1\atopwithdelims ()2}+\cdots +{n_m\atopwithdelims ()2}-{N\atopwithdelims ()2}}}{\prod _{j=1}^m(aq/e_j,q/e_j;q)_{n_j}e_j^{n_j}}, \end{aligned}$$

where $$N=n_1+\cdots +n_m$$.

Other special cases of (2.2) that we require are the following (see [3, (1.9.9), (1.9.12)]):

2.4 $$\begin{aligned} \sum _{k=0}^N\frac{(q^{-N},b_1q^{n_1},\dots ,b_m q^{n_m};q)_k}{(q,b_1,\ldots ,b_m;q)_k}q^k =(-1)^N\frac{(q;q)_N \,b_1^{n_1}\cdots b_m^{n_m}}{(b_1;q)_{n_1}\cdots (b_m;q)_{n_m}}q^{{n_1\atopwithdelims ()2}+\cdots +{n_m\atopwithdelims ()2}},\nonumber \\ \quad \end{aligned}$$

where $$N=n_1+\cdots +n_m$$, and

2.5 $$\begin{aligned} \sum _{k=0}^N\frac{(q^{-N},b_1q^{n_1},\dots ,b_m q^{n_m};q)_k}{(q,b_1,\ldots ,b_m;q)_k} =(-1)^N\frac{(q;q)_N \,q^{-{N+1\atopwithdelims ()2}}}{(b_1;q)_{n_1}\cdots (b_m;q)_{n_m}}, \end{aligned}$$

where $$N\geqslant n_1+\cdots +n_m$$.

## A parametric generalization and proof of Theorem 1.1

We first give the following result.

### Lemma 3.1

Let $d\geqslant 3$ be an odd integer and let $n\equiv 1\pmod {d}$ be a positive integer. Then, for any integer *A*, modulo $\Phi _n(q)^2$,

3.1 $$\begin{aligned} (q^{2+An},q^{d+An};q^d)_{(2n-2)/d}\equiv q^{2An(n-1)/d} (q^2,q^d;q^d)_{(2n-2)/d} \pmod {\Phi _n(q)^2}.\qquad \end{aligned}$$

### Proof

We shall prove the following parametric generalization of (3.1):

3.2 $$\begin{aligned} (aq^2,aq^d;q^d)_{(2n-2)/d}\equiv a^{2(n-1)/d}(q^2,q^d;q^d)_{(2n-2)/d} \pmod {(1-a)(1-aq^{2n})}. \qquad \end{aligned}$$

It is clear that both sides of (3.2) are equal for $a=1$. This means that the congruence (3.2) holds modulo $1-a$. Further, for $a=q^{-2n}$ the left-hand side of (3.2) is equal to $(q^{2-2n},q^{d-2n};q^d)_{(2n-2)/d}$. But

$$\begin{aligned} (q^{2-2n};q^d)_{(2n-2)/d}&=(1-q^{2-2n})(1-q^{2-2n+d})\cdots (1-q^{-d}) \\&=q^{-(d+2n-2)(n-1)/d}(1-q^{2n-2})(1-q^{2n-2-d})\cdots (1-q^d) \\&=q^{-(d+2n-2)(n-1)/d}(q^d;q^d)_{(2n-2)/d}, \end{aligned}$$

and similarly,

$$\begin{aligned} (q^{d-2n};q^d)_{(2n-2)/d} =q^{-(2+2n-d)(n-1)/d}(q^2;q^d)_{(2n-2)/d}. \end{aligned}$$

This proves that for $a=q^{-2n}$ both sides of (3.2) are also equal, and so the congruence (3.2) is true modulo $1-aq^{2n}$. Since $1-a$ and $1-aq^{2n}$ are relatively prime polynomials, we obtain (3.2).

Let $a=q^{An}$ in (3.2). Noticing that both $1-q^{An}$ and $1-q^{(A+2)n}$ contain the factor $\Phi _n(q)$, we arrive at the *q*-congruence (3.1). $\square$

We now give a parametric generalization of Theorem 1.1.

### Theorem 3.2

Let $d\geqslant 3$ be an odd integer and let $n\equiv 1\pmod {d}$ be a positive integer. Then, modulo $\Phi _n(q)(1-aq^n)(a-q^n)$,

3.3 $$\begin{aligned}&\sum _{k=0}^{n-1}[2dk+d-2] \frac{(a^{d-2}q^{d-2}, a^{d-4}q^{d-2},\ldots , aq^{d-2};q^d)_k}{(a^{d-2}q^d, a^{d-4}q^d,\ldots ,aq^d;q^d)_k}\nonumber \\&\qquad \times \frac{(a^{2-d}q^{d-2}, a^{4-d}q^{d-2},\ldots , a^{-1}q^{d-2};q^d)_k (q^{d-2};q^d)_k}{(a^{2-d}q^d, a^{4-d}q^d,\ldots , a^{-1}q^d;q^d)_k (q^d;q^d)_k } \nonumber \\&\quad \equiv \frac{[(d-2)n](q^{d-2},q^d;q^d)_{(d-2)(n-1)/d} \,q^{-(d-1)^2(n-1)/(2d)}}{(q,q^{d-1};q^d)_{(n-1)/d}\prod _{j=1}^{(d-3)/2} (q^{d+(d-2j-2)n},q^{2+(d-2j-2)n};q^d)_{(2n-2)/d}}. \end{aligned}$$

### Proof

Since $\gcd (d,n)=1$, none of the numbers $d,2d,\ldots , (n-1)d$ are divisible by *n*. Therefore, the denominators of the left-hand side of (3.3) do not have the factor $1-aq^n$ nor $1-a^{-1}q^n$. Thus, for $a=q^{-n}$ or $a=q^n$, the left-hand side of (3.3) can be written as

3.4 $$\begin{aligned}&\sum _{k=0}^{(d-2)(n-1)/d}[2dk+d-2] \frac{(q^{d-2-(d-2)n}, q^{d-2-(d-4)n},\ldots ,q^{d-2-n};q^d)_k }{(q^{d-(d-2)n}, q^{d-(d-4)n},\ldots , q^{d-n};q^d)_k } \nonumber \\&\quad \qquad \times \frac{(q^{(d-2)n+d-2}, q^{(d-4)n+d-2},\ldots ,q^{n+d-2};q^d)_k (q^{d-2};q^d)_k}{(q^{(d-2)n+d}, q^{(d-4)n+d},\ldots , q^{n+d};q^d)_k (q^d;q^d)_k }, \end{aligned}$$

where we have used $(q^{d-2-(d-2)n};q^d)_k=0$ for $k>(d-2)(n-1)/d$. Specializing the parameters in (2.3) by $N=(d-2)(n-1)/d$, $q\mapsto q^d$, $a=q^{d-2}$, $m=(d-1)/2$, $e_j=q^{d-2-(d-2j-2)n}$ ($1\leqslant j\leqslant m-1$), $e_m=q^{d-1}$, $n_1=\cdots =n_{m-1}=(2n-2)/d$ and $n_m=(n-1)/d$ and noticing $N=n_1+\cdots +n_m$, we see that (3.4) is equal to the right-hand side of (3.3), where we have used the relation

$$\begin{aligned}{}[d-2](q^{2d-2};q^d)_{(d-2)(n-1)/d}=[(d-2)n](q^{d-2};q^d)_{(d-2)(n-1)/d}. \end{aligned}$$

This proves that (3.3) holds modulo $(1-aq^n)(a-q^n)$.

For $M=(d-2)(n-1)/d$, by Lemma 2.1, we can easily verify that

$$\begin{aligned}&[2d(M-k)+d-2]\frac{(a^{d-2}q^{d-2}, a^{d-4}q^{d-2},\ldots , aq^{d-2};q^d)_{M-k}}{(a^{d-2}q^d, a^{d-4}q^d,\ldots ,aq^d;q^d)_{M-k}}\\&\qquad \times \frac{(a^{2-d}q^{d-2}, a^{4-d}q^{d-2},\ldots ,a^{-1}q^{d-2};q^d)_{M-k} (q^{d-2};q^d)_{M-k}}{(a^{2-d}q^d, a^{4-d}q^d,\ldots , a^{-1}q^d;q^d)_{M-k} (q^d;q^d)_{M-k} } \\&\quad \equiv -[2dk+d-2]\frac{(a^{d-2}q^{d-2}, a^{d-4}q^{d-2},\ldots , aq^{d-2};q^d)_k}{(a^{d-2}q^d, a^{d-4}q^d,\ldots ,aq^d;q^d)_k}\\&\qquad \times \frac{(a^{2-d}q^{d-2}, a^{4-d}q^{d-2},\ldots , a^{-1}q^{d-2};q^d)_k (q^{d-2};q^d)_k}{(a^{2-d}q^d, a^{4-d}q^d,\ldots , a^{-1}q^d;q^d)_k (q^d;q^d)_k } \pmod {\Phi _n(q)}. \end{aligned}$$

It is now evident that the sum of the *k*-th and $(M-k)$-th summands on the left-hand side of (3.3) vanishes modulo $\Phi _n(q)$. Since $[(d-2)n]\equiv 0\pmod {\Phi _n(q)}$, we conclude that the partial sum of the left-hand side of (3.3) for *k* up to $(d-2)(n-1)/d$ is congruent to 0 modulo $\Phi _n(q)$. Further, for any *k* satisfying $(d-2)(n-1)/d<k\leqslant n-1$, we have $(q^{d-2};q^d)_k/(q^d;q^d)_k\equiv 0\pmod {\Phi _n(q)}$. This proves the *q*-congruence (3.3). $\square$

### Proof of Theorem 1.1

In view of $\gcd (n,d)=1$, the factors involving *a* in the denominators of the left-hand side of (3.3) are all relatively prime to $\Phi _n(q)$ when $a=1$. On the other hand, the polynomial $(1-aq^n)(a-q^n)$ contains the factor $\Phi _n(q)^2$ when $a=1$. Hence, taking $a=1$ in (3.3), we obtain

$$\begin{aligned}&\sum _{k=0}^{n-1}[2dk+d-2]\frac{(q^{d-2};q^d)_k^d}{(q^d;q^d)_k^d} \nonumber \\&\quad \equiv \frac{[(d-2)n](q^{d-2},q^d;q^d)_{(d-2)(n-1)/d}\, q^{-(d-1)^2(n-1)/(2d)}}{(q,q^{d-1};q^d)_{(n-1)/d}\prod _{j=1}^{(d-3)/2} (q^{d+(d-2j-2)n},q^{2+(d-2j-2)n};q^d)_{(2n-2)/d}} \pmod {\Phi _{n}(q)^3}. \end{aligned}$$

Furthermore, applying Lemma 3.1$(d-3)/2$ times, modulo $\Phi _n(q)^2$, we have

$$\begin{aligned} \prod _{j=1}^{(d-3)/2}(q^{2+(d-2j-2)n},q^{d+(d-2j-2)n};q^d)_{(2n-2)/d} \equiv (q^2,q^d;q^d)_{(2n-2)/d}^{(d-3)/2}\,q^{(d-3)^2n(n-1)/(2d)}. \end{aligned}$$

This, together with $[(d-2)n]\equiv 0\pmod {\Phi _n(q)}$, proves (1.6). $\square$

## A parametric generalization and proof of Theorem 1.2

As before, we first give a parametric generalization of Theorem 1.2.

### Theorem 4.1

Let $d\geqslant 3$ be an odd integer and let $n\equiv 2\pmod {d}$ be a positive integer. Then, modulo $(1-aq^n)(a-q^n)$,

4.1 $$\begin{aligned}&\sum _{k=0}^{n-1}[2dk+d-2]\frac{(a^{d-1}q^{d-2}, a^{d-3}q^{d-2},\ldots , a^2q^{d-2};q^d)_k}{(a^{d-2}q^d, a^{d-4}q^d,\ldots ,aq^d;q^d)_k}\nonumber \\&\qquad \times \frac{(a^{1-d}q^{d-2}, a^{3-d}q^{d-2},\ldots , a^{-2}q^{d-2};q^d)_k (q^{d-2};q^d)_k}{(a^{2-d}q^d, a^{4-d}q^d,\ldots , a^{-1}q^d;q^d)_k (q^d;q^d)_k } \nonumber \\&\quad \equiv -\frac{(q^d;q^d)_{(dn-n-d+2)/d}(1+q^{n^2(d-1)/2}) q^{-d{(dn-n+2)/d\atopwithdelims ()2}} }{(1-q)(a^{d-2}q^d,a^{d-4}q^d,\ldots , a^{4-d}q^d,a^{2-d}q^d;q^d)_{(n-2)/d}} . \end{aligned}$$

### Proof

For $a=q^{-n}$ or $a=q^n$, the left-hand side of (4.1) can be written as

4.2 $$\begin{aligned}&\sum _{k=0}^{(dn-n-d+2)/d}[2dk+d-2]\frac{(q^{d-2-(d-1)n}, q^{d-2-(d-3)n},\ldots ,q^{d-2-2n};q^d)_k }{(q^{d-(d-2)n}, q^{d-(d-4)n},\ldots , q^{d-n};q^d)_k } \nonumber \\&\quad \qquad \times \frac{(q^{(d-1)n+d-2}, q^{(d-3)n+d-2},\ldots , q^{2n+d-2};q^d)_k(q^{d-2};q^d)_k}{(q^{(d-2)n+d}, q^{(d-4)n+d},\ldots , q^{n+d};q^d)_k (q^d;q^d)_k }, \end{aligned}$$

where we have used $(q^{d-2-(d-1)n};q^d)_k=0$ for $k>(dn-n-d+2)/d$. Since

$$\begin{aligned}{}[2dk+d-2]&=\frac{1-q^{2dk+d-2}}{1-q} \\&=\frac{1-q^{(d-2)/2}}{1-q}\frac{(q^{d+(d-2)/2};q^d)_k}{(q^{(d-2)/2};q^d)_k}q^{dk+(d-2)/2}\\&\quad +\frac{1-q^{(d-2)/2}}{1-q}\frac{(q^{d+(d-2)/2};q^d)_k}{(q^{(d-2)/2};q^d)_k}, \end{aligned}$$

by (2.4) and (2.5), the summation (4.2) is equal to

$$\begin{aligned}&\frac{q^{(d-2)/2}(-1)^{(dn-n-d+2)/d}(q^d;q^d)_{(dn-n-d+2)/d} \,q^{(d-2)/2+(n-2)(d-1)}}{(1-q)(q^{d-(d-2)n}, q^{d-(d-4)n},\ldots , q^{d+(d-4)n},q^{d+(d-2)n};q^d)_{(n-2)/d} } q^{d(d-1){(n-2)/d\atopwithdelims ()2}} \\&\quad \qquad +\frac{(-1)^{(dn-n-d+2)/d}(q^d;q^d)_{(dn-n-d+2)/d} \,q^{-d{(dn-n+2)/d\atopwithdelims ()2}}}{(1-q)(q^{d-(d-2)n}, q^{d-(d-4)n}, \ldots , q^{d+(d-4)n},q^{d+(d-2)n};q^d)_{(n-2)/d} }, \end{aligned}$$

which is just the $a=q^{-n}$ or $a=q^n$ case of the right-hand side of (4.1). This proves the theorem. $\square$

### Proof of Theorem 1.2

Since $\gcd (n,d)=1$, the factors related to *a* in the denominators of the left-hand side of (4.1) are relatively prime to $\Phi _n(q)$ for $a=1$. Thus, letting $a=1$ in (4.1), we conclude that (1.8) is true modulo $\Phi _n(q)^2$. $\square$

## A parametric generalization and proof of Theorem 1.3

We need the following result.

### Lemma 5.1

Let $d\geqslant 3$ be an integer and let $n\equiv 1\pmod {d}$ be a positive integer. Then, for any integer *A*, modulo $\Phi _n(q)^2$,

5.1 $$\begin{aligned} (q^{1+An},q^{d+An};q^d)_{(n-1)/d}\equiv q^{An(n-1)/d} (q,q^d;q^d)_{(n-1)/d} \pmod {\Phi _n(q)^2}. \end{aligned}$$

### Proof

The proof is similar to that of Lemma 3.1. Here we only give its parametric version of (5.1):

$$\begin{aligned} (aq,aq^d;q^d)_{(n-1)/d}\equiv a^{(n-1)/d}(q,q^d;q^d)_{(n-1)/d} \pmod {(1-a)(1-aq^n)}. \end{aligned}$$

$$\square $$

We have the following parametric generalization of Theorem 1.3.

### Theorem 5.2

Let $d\geqslant 3$ be an integer and let $n\equiv 1\pmod {d}$ be a positive integer. Then, modulo $\Phi _n(q)(1-aq^n)(a-q^n)$,

5.2 $$\begin{aligned}&\sum _{k=0}^{M}[2dk+d-1]\frac{(a^{d-1}q^{d-1}, a^{d-2}q^{d-1},\ldots , aq^{d-1},q^{d-1};q^d)_k}{(a^{d-1}q^d, a^{d-2}q^d,\ldots ,aq^d,q^d;q^d)_k}\nonumber \\&\qquad \times \frac{(a^{1-d}q^{d-1}, a^{2-d}q^{d-1},\ldots , a^{-1}q^{d-1},q^{d-1};q^d)_k }{(a^{1-d}q^d, a^{2-d}q^d,\ldots , a^{-1}q^d,q^d;q^d)_k } \nonumber \\&\quad \equiv [(d-1)n]\frac{(q^{d-1},q^d;q^d)_{(d-1)(n-1)/d}\,q^{-(d-1)(n-1)/2}}{\prod _{j=1}^{d-1}(q^{d+(d-j-1)n},q^{1+(d-j-1)n};q^d)_{(n-1)/d}}, \end{aligned}$$

where $$M=(d-1)(n-1)/d$$ or $$M=n-1$$.

### Proof

For $a=q^{-n}$ or $a=q^n$, the left-hand side of (5.2) can be written as

5.3 $$\begin{aligned}&\sum _{k=0}^{(d-1)(n-1)/d}[2dk+d-1]\frac{(q^{d-1-(d-1)n}, q^{d-1-(d-2)n}, \ldots ,q^{d-1-n},q^{d-1};q^d)_k }{(q^{d-(d-1)n}, q^{d-(d-2)n},\ldots , q^{d-n},q^d;q^d)_k } \nonumber \\&\quad \qquad \times \frac{(q^{(d-1)n+d-1}, q^{(d-2)n+d-1},\ldots ,q^{n+d-1},q^{d-1};q^d)_k }{(q^{(d-1)n+d}, q^{(d-2)n+d},\ldots , q^{n+d}, q^d;q^d)_k }, \end{aligned}$$

where we have utilized $(q^{d-1-(d-1)n};q^d)_k=0$ for $k>(d-1)(n-1)/d$. Performing the parameter substitutions $N=(d-1)(n-1)/d$, $q\mapsto q^d$, $a=q^{d-1}$, $m=d-1$, $e_j=q^{d-1-(d-j-1)n}$ ($1\leqslant j\leqslant m$), $n_1=\cdots =n_{m}=(n-1)/d$ in (2.3), we see that (5.3) is equal to the right-hand side of (5.2), where we have used the relation

$$\begin{aligned}{}[d-1](q^{2d-1};q^d)_{(d-1)(n-1)/d}=[(d-1)n](q^{d-1};q^d)_{(d-1)(n-1)/d}. \end{aligned}$$

This proves that (5.2) is true modulo $(1-aq^n)(a-q^n)$.

For $M=(d-1)(n-1)/d$, by Lemma 2.1, we can easily verify that

$$\begin{aligned}&[2d(M-k)+d-1]\frac{(a^{d-1}q^{d-1}, a^{d-2}q^{d-1},\ldots , aq^{d-1},q^{d-1};q^d)_{M-k}}{(a^{d-1}q^d, a^{d-2}q^d,\ldots ,aq^d,q^d;q^d)_{M-k}}\\&\qquad \times \frac{(a^{1-d}q^{d-1}, a^{2-d}q^{d-1},\ldots , a^{-1}q^{d-1},q^{d-1};q^d)_{M-k} }{(a^{1-d}q^d, a^{2-d}q^d,\ldots , a^{-1}q^d,q^d;q^d)_{M-k} } \\&\quad \equiv -[2dk+d-1]\frac{(a^{d-1}q^{d-1}, a^{d-2}q^{d-1},\ldots , aq^{d-1},q^{d-1};q^d)_k}{(a^{d-1}q^d, a^{d-2}q^d,\ldots ,aq^d,q^d;q^d)_k}\\&\qquad \times \frac{(a^{1-d}q^{d-1}, a^{2-d}q^{d-1},\ldots , a^{-1}q^{d-1},q^{d-1};q^d)_k }{(a^{1-d}q^d, a^{2-d}q^d,\ldots , a^{-1}q^d,q^d;q^d)_k } \pmod {\Phi _n(q)}. \end{aligned}$$

Since $[(d-2)n]\equiv 0\pmod {\Phi _n(q)}$, we conclude that (5.2) holds modulo $\Phi _n(q)$. Further, for any *k* in the range $(d-1)(n-1)/d<k\leqslant n-1$, we have $(q^{d-1};q^d)_k/(q^d;q^d)_k\equiv 0\pmod {\Phi _n(q)}$. This means that the *q*-congruence (5.2) also holds modulo $\Phi _n(q)$ for $M=n-1$. $\square$

### Proof of Theorem 1.3

Since $\gcd (n,d)=1$, the factors related to *a* in the denominators of the left-hand side of (5.2) are coprime with $\Phi _n(q)$ for $a=1$. Thus, letting $a=1$ in (5.2), we conclude that

5.4 $$\begin{aligned}&\sum _{k=0}^{M}[2dk+d-1]\frac{(q^{d-1};q^d)_k^{2d}}{(q^d;q^d)_k^{2d}} \nonumber \\&\quad \qquad \equiv [(d-1)n]\frac{(q^{d-1},q^d;q^d)_{(d-1)(n-1)/d}\, q^{-(d-1)(n-1)/2}}{\prod _{j=1}^{d-1} (q^{d+(d-j-1)n},q^{1+(d-j-1)n};q^d)_{(n-1)/d}}\pmod {\Phi _n(q)^3}, \end{aligned}$$

Moreover, by repeatedly using Lemma 5.1$d-1$ times, we obtain

$$\begin{aligned}&\prod _{j=1}^{d-1}(q^{d+(d-j-1)n},q^{1+(d-j-1)n};q^d)_{(n-1)/d} \\&\quad \equiv (q,q^d;q^d)_{(n-1)/d}^{d-1}\, q^{(d-1)(d-2)n(n-1)/(2d)} \pmod {\Phi _n(q)^2}. \end{aligned}$$

Substituting this into (5.4), we see that the *q*-congruence (1.10) is true modulo $\Phi _n(q)^3$. Finally, the validity of (1.10) modulo [*n*] follows from [7, Lemma 4.1]. $\square$

## Concluding remarks

The present authors [7, Theorem 1.1] gave the following result: Let *d*, *n*, *r* be integers satisfying $d\geqslant 2$, $r\leqslant d-2$ (in particular, *r* may be negative), and $n\geqslant d-r$, such that *d* and *r* are coprime, and $n\equiv -r\pmod {d}$. Then

6.1 $$\begin{aligned}&\sum _{k=0}^{M}[2dk+r]\frac{(q^r;q^d)_k^4}{(q^d;q^d)_k^4}q^{(d-2r)k} \nonumber \\&\quad \equiv {\left\{ \begin{array}{ll} 0 \pmod {[n]\Phi _n(q)^3} &{}\text {if }d=2,\\ q^{r(n+r-dn)/d}\dfrac{(q^{2r};q^d)_{(dn-n-r)/d}}{(q^d;q^d)_{(dn-n-r)/d}}[dn-n] \pmod {[n]\Phi _n(q)^3} &{}\text {if }d\geqslant 3, \end{array}\right. } \end{aligned}$$

where $$M=(dn-n-r)/d$$ or $$n-1$$.

Here we point out that (6.1) is also true for $d\geqslant 3$ and $r=d-1$ (and thus $n\equiv 1\pmod {d}$). This is because the proof of [7, Theorem 3.1] is also valid for $r=d-1$, since $2r+2n\leqslant dn$ still holds for $n\geqslant d+1$ in this case [(6.1) clearly holds for $n=1$].
